# Maternal HIV Infection as a Risk Factor for Primary Epstein-Barr Virus Infection in Kenyan Infants

**DOI:** 10.3389/fonc.2021.805145

**Published:** 2022-01-12

**Authors:** Gabriela Samayoa-Reyes, Sidney O. Ogolla, Ibrahim I. Daud, Conner Jackson, Katherine R. Sabourin, Arlene Dent, Rosemary Rochford

**Affiliations:** ^1^ Department of Immunology and Microbiology, University of Colorado, School of Medicine, Aurora, CO, United States; ^2^ Center for Global Health Research, Kenya Medical Research Institute, Kisumu, Kenya; ^3^ Center for Global Health and Diseases, Case Western Reserve University, Cleveland, OH, United States

**Keywords:** Epstein - Barr virus, HIV - human immunodeficiency virus, Kenya, Burkitt lymphoma (BL), EBV transmission, HIV-exposed uninfected (HEU) infants, HIV- unexposed uninfected (HUU) infants

## Abstract

Human immunodeficiency virus (HIV) infection is known to be associated with EBV shedding in saliva suggesting an increased risk of EBV transmission to infants born to mothers with HIV at an earlier age. In this study we investigated (i) whether maternal HIV status was a risk factor for EBV in blood at delivery or for shedding in saliva and breast milk of 6- and 10-weeks post-partum mothers, (ii) if there was a difference in EBV strains shed between HIV+ and HIV- mothers, and (iii) if maternal HIV status was a determinant of EBV viral load in their infants. Samples were collected as part of a prospective cohort study that followed HIV-positive (HIV+) and HIV-negative (HIV-) pregnant women in Western Kenya through delivery and post-partum period. EBV viral load in blood was found to be significantly higher in mothers with HIV (p-value = 0.04). Additionally, a statistically significant difference was observed between EBV viral load in saliva samples and HIV status where HIV+ mothers had a higher EBV viral load in saliva at 6-weeks post-partum compared to HIV- mothers (p-value < 0.01). The difference in EBV shedding in breast milk was not found to be statistically significant. Furthermore, no difference in frequency of EBV strain was attributable to HIV- or HIV+ mothers. Interestingly, we found that infants born to HIV+ mothers had a higher EBV viral load at the time of their first EBV detection in blood than infants born to HIV- mothers and this was independent of age at detection. Overall, our study suggests that HIV infected mothers shed more virus in saliva than HIV-negative mothers and infants born to HIV+ mothers were at risk for loss of control of primary EBV infection as evidenced by higher EBV viral load following primary infection.

## Introduction

Epstein-Barr virus (EBV) is a human gammaherpesvirus associated with many human malignancies including the pediatric malignancy, endemic Burkitt lymphoma (eBL). Early age of EBV infection is thought to be a risk factor for the development of BL (1). Consistent with this model, we have previously found that infants living in a high BL incidence region of Western Kenya (2) were infected at less than 6 months of age and those infected early in life had poor control of EBV infection (3). In the context of HIV infection, others have found that HIV+ infants are infected earlier in life with EBV, and have higher EBV viral loads compared to HIV- infants (4). Together this data points towards the mother, who is the primary caregivers of infants as a potential source of EBV infection for their infants.

EBV infects *via* the oropharyngeal route and is transmitted through saliva (5, 6). Infectious EBV virus has been detected in breast milk of mothers and can be another potential source of infection to infants (7). In cross-sectional studies of EBV shedding in the saliva in healthy adult populations, the prevalence of detection ranged from reporting that EBV-infected individual always sheds virus (8) while others have found a range of frequency from those that never shed the virus to always shedding the virus (9, 10). Prior to the introduction of antiretroviral therapy (ART), EBV was frequently detected and viral load was higher in saliva of HIV+ individuals compared to HIV- individuals (11, 12). In a study in Cameroon, EBV shedding was more frequent and EBV viral loads were higher in HIV+ compared to HIV- individuals but no data on treatment status was available (13). In contrast, in patients on ART in Uganda, Matrajt et al. (14) reported higher rates of EBV shedding in HIV+ compared to HIV- mothers but no significant difference was observed in EBV viral load in saliva.

There are two known strains of EBV, Type 1 (EBV-1) and Type 2 (EBV-2), primarily classified based on divergence in the EBV nuclear antigen 2 (EBNA-2) and EBV nuclear antigen 3 (EBNA-3a, EBNA-3b, and EBNA-3c) genes (15, 16, 1; 17). EBV-1 is the dominant strain found worldwide. EBV-2 is also found around the world, but high prevalence is limited to certain populations including HIV+ individuals and in sub-Saharan Africa where BL is endemic (15, 18). Based on data from a transformation assay using saliva samples, shedding of both EBV-1 and EBV-2 occurred rarely, if at all (9). But we found evidence of co-infection in peripheral blood of healthy infants in Kenya (18) and Van Baarle et al. (19) found that co-infection was more common in HIV+ compared to HIV- individuals.

In sub-Saharan Africa, highly active antiretroviral therapy (HAART) and limited perinatal ART interventions greatly reduced the number of HIV infected newborns leaving a growing population of HIV-exposed *in utero* but uninfected (HEU) infants. Several studies have shown that these HIV HEU infants have increased morbidity and mortality (20–24). The underlying mechanism is likely multifactorial.

To date, only a few studies have investigated the impact of infant HIV infection on EBV infection. An earlier study comparing HIV+ and HIV- infants born to HIV+ mothers (25) reported that early age of EBV infection is common in infants born to HIV+ mothers. Another study looked at the impact of infant HIV infection on primary EBV infection (4) and found that infant HIV infection was associated with early age of EBV infection and higher EBV viral loads. Similarly, Gumbo et al. (26) looked at EBV acquisition in HIV+ Zimbabwean infants and reported that EBV co-infection occurred earlier than anticipated and identified congenital EBV infection. More recently, Montoya-Ferrer et al. (27) studied the onset of EBV infection in HEU infants. They found that young maternal age and detectable levels of HIV RNA in plasma were associated EBV infection during the first year of life, but no comparison between HEU infants and HIV-unexposed uninfected (HUU) infants was made.

In this study, we evaluated whether maternal HIV status had an effect on EBV viral load in HEU and HUU infants and if mothers might be the primary source of infection for infants in a cohort previously evaluated from Western Kenya (28, 29). We analyzed EBV prevalence, EBV viral load, and EBV type in HIV+ and HIV- mothers at time of delivery as well as in saliva and breast milk of mother’s post-partum and we evaluated EBV viral load and EBV type in their infants. We investigated (i) whether there was a difference in both frequency of EBV detection and EBV viral load in HIV+ compared to HIV- mothers, (ii) if co-infection with EBV-1 and EBV-2 was more common in HIV+ mothers, and (iii) if maternal EBV type in saliva or breast milk was similar to their infants, and if the HIV status of the mother have any effect on infant EBV viral load.

## Materials and Methods

### Study Site and Populations

Participants in this study were recruited from Chulaimbo County Hospital, which serves a rural population in Kisumu County, Kenya, as part of the **C**hulaimbo **A**ntenatal **P**ost-Natal (CHAP) cohort study conducted from 2011 to 2015. Details of recruitment and follow-up have been previously described (28, 29). Briefly, pregnant women, both HIV+ and HIV-, were recruited from a malaria-endemic high-risk region of Kenya. HIV+ mothers were enrolled at the Academic Model Providing Access To Healthcare (AMPATH) site that provided clinical services and medications for HIV+ patients and their families. At AMPATH sites, all HIV+ pregnant women receive HAART (Lamivudine + Zidovudine (Hb>8) or Tenofovir (Hb<8) + Nevirapine (CD4<250) or Lopinavir/r (CD4>250). Pregnant women were enrolled at their first prenatal visit (typically during the second trimester) and longitudinally followed throughout pregnancy (up to 4 prenatal visits). Following delivery, mothers who delivered at a health facility, had no blood transfusion within 24 hours of delivery and had a normal vaginal delivery with term and liveborn singletons were followed during the post-partum period. Where possible breast milk and saliva samples were collected at approximately 6- and 10-weeks post-partum.

Written informed consent was obtained from each study participant. Approval for this study was obtained from the Scientific and Ethical Review Unit (SERU) at the Kenya Medical Research Institute (KEMRI), Colorado Multiple Institutional Review Board (COMIRB), and SUNY Upstate Medical University (where the study was initiated) ethical review boards.

### Blood Sample Collection and Processing

At delivery, maternal venous blood samples were collected in EDTA microtainers (Becton Dickinson). Infant blood was collected by finger or heel prick into EDTA microtainers. Blood was centrifuged to isolate the plasma fraction from the cell pellet, and an equivalent volume of phosphate-buffered saline was added to replace plasma volume removed. Plasma and blood samples were collected and stored at -80°C. All samples were processed within 5 hours of collection at the laboratory facilities at the Center for Global Health Research (CGHR) at KEMRI. DNA was extracted from up to 200 μL of blood using a Qiagen DNAeasy kit (Qiagen) according to the manufacturer’s protocol.

### Breast Milk Specimen Collection and Processing

Breast milk specimens (3-5mL) were collected and processed as previously reported (7). Genomic DNA was extracted using the QiaAmp DNA Mini Kit (Qiagen, Valencia, California) in accordance with the manufacturer’s protocol. DNA purity and quantity were assessed with a NanoDrop 2000 spectrophotometer (Thermo Scientific, Wilmington, Delaware).

### Saliva Specimen Collection and Processing

Saliva specimens were collected as described (13). Samples were centrifuged and stored at -80°C. DNA was extracted from saliva using the NucleoSpin DNA RapidLyse (Takara, Mountain View, California) in accordance with the manufacturer’s protocol. DNA purity and quantity were assessed with a NanoDrop 2000 spectrophotometer (Thermo Scientific, Wilmington, Delaware).

### Measurement of EBV Viral Load by qPCR in Venous Blood, Breast Milk, and Saliva Specimens

EBV DNA was detected and quantified by quantitative polymerase chain reaction (qPCR) as described previously (3, 30). EBV load was expressed as copies/mL of breast milk or saliva or as copies/µg of DNA for blood.

### EBV Typing

The EBNA-3C region was amplified in DNA extracted from saliva by qPCR designed to distinguish between EBV-1 and EBV-2 in the EBNA-3C region as previously described (7). All reactions were performed with the TaqMan Advance Fast Master Mix (Applied Biosystems, Waltham, MA).

### Statistical Analysis

Participant characteristics were analyzed using Fisher’s exact test or chi square tests for categorical data and either unequal variance t-tests or Mann-Whitney tests for continuous data, as appropriate. Analyses of EBV load in maternal venous blood utilized a zero-inflated modeling approach with the MAST package (version 1.16.0) (31). For saliva and breastmilk data, the proportion of responders was analyzed with a two-sample test of proportions; of the responders, Mann-Whitney tests were used for pairwise testing. When evaluating changes in saliva and breastmilk over time from 6 to 10 weeks, a pairwise Wilcoxon signed rank exact test was used to account for the repeated measures. For comparisons of more than two groups, an ANOVA or Kruskal Wallis test (in the event of ANOVA assumption violations) was used followed by pairwise Mann-Whitney tests with Bonferroni correction if justified based on ANOVA or Kruskal Wallis results. To evaluate paired (data at 6 and 10 weeks) differences between time points and by HIV status, a Friedman Rank Sum Test was used. Statistical significance was defined by an alpha of < 0.05. All analyses were conducted with R (version 4.0.2) (32).

## Results

### Characteristics of Study Participants

**Figure 1:**
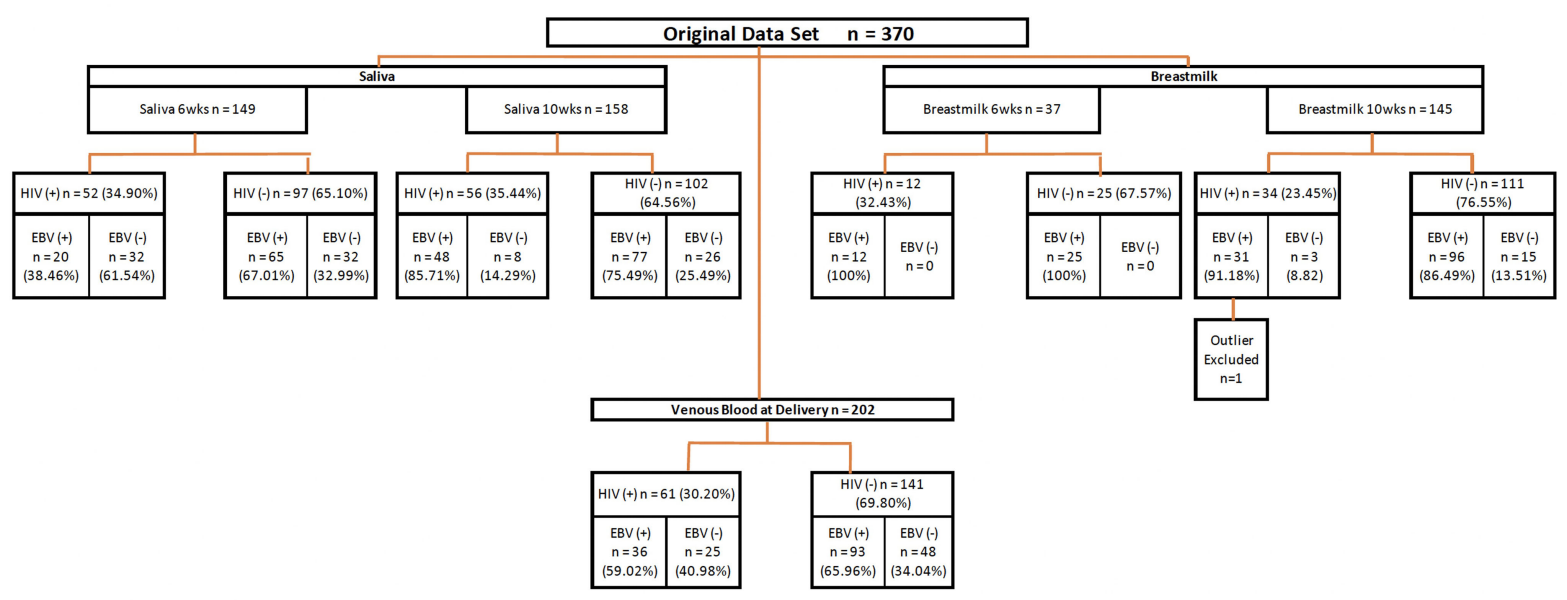
Diagram of study procedures. Flow chart summarizing the source of maternal samples analyzed. Wks, weeks; EBV, Epstein-Barr Virus; HIV, Human immunodeficiency virus.

The study participants were described previously (28, 29). Briefly, the cohort consisted of 370 women who were followed from their first antenatal visit through delivery, and the post-partum period. Women that had delivery venous blood, saliva or breast milk samples available at 6 or 10-weeks post-partum were included in our analyses (n=243). **Figure 1** illustrates the numbers of maternal samples analyzed for EBV studies and their HIV status. The median age of HIV- women was 20 years, and the HIV+ mothers had a median age of 27 years. (**Table 1**). 45.7% of the HIV- mothers were primigravidae as compared to 15.2% of the HIV+. No differences in education were identified between HIV+ and HIV- mothers.

**Table 1:** Descriptive Statistics of Study Participants.

	Mothers without HIV	Mothers with HIV	p-value
	(N = 265)	(N = 105)	
**Age**			
Median [Q1, Q3]	20.0 [18.0, 24.0]	27.0 [22.0, 32.0]	<0.01
**Primigravidae**			
No	53.6% (142)	84.8% (89)	<0.01
Yes	45.7% (121)	15.2% (16)	
Missing	0.8% (2)	0% (0)	
**Education**			
None or Lower Primary School	14.0% (37)	12.4% (13)	0.94
Upper Primary or Secondary School	82.3% (218)	83.8% (88)	
Adult Education, Village Polytechnic, Other	3.8% (10)	3.8% (4)	

### Cell-Associated EBV and Viral Shedding in Saliva Is More Frequent in HIV- Mothers but EBV Load Is Higher in HIV+ Mothers

**Figure 2:**
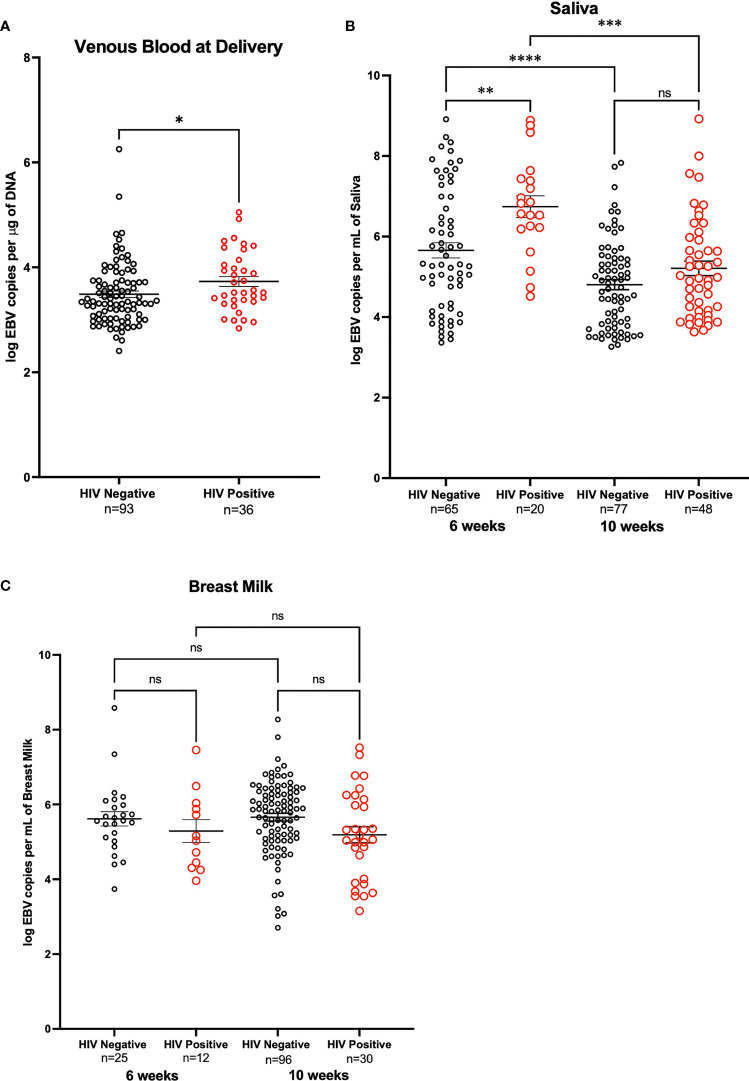
EBV Viral load in HIV negative and positive mothers. **(A)** Maternal venous blood cell pellet at time of delivery. Analysis made on a zero-inflated model. DNA from venous blood from HIV- mothers (n=141) and HIV+ mothers (n=61) was tested for EBV viral load, only those who had detectable EBV are shown. HIV-mothers had a mean log EBV copies per μg of DNA of 3.49 and HIV+ mothers had a mean log EBV copies per μg of DNA of 3.73 (p-value = 0.04) **(B)** DNA from saliva from mother at 6-weeks post-partum that were HIV- (n=97) and HIV+ (n=52) (p-value < 0.01), as well as mothers at 10-weeks post-partum that were HIV- (n=103) and HIV+ (n=56) were tested for EBV shedding, only those that are positive shedders are shown. Comparison across time points account for the paired nature of the data and were analyzed with Wilcoxon signed rank exact test; differences among HIV+ (p-value < 0.01) and HIV- (p-value < 0.01) were observed. **(C)** Breast Milk DNA from mothers at 6- weeks post-partum both HIV- (n=25) and HIV+ (n=12) and breast milk of mothers 10-weeks post-partum HIV- (n=111) and HIV+ (n=34) were analyzed for shedding of EBV. Only mothers in which shedding was detected are shown. *P < 0.05, **P < 0.005, ***P < 0.0005, ****P < 0.0001, ns, not significant.

EBV was quantified by qPCR using DNA extracted from maternal venous blood at delivery (n=202). The proportion of detectable EBV in blood was lower for HIV+ mothers (59.02%) as compared to HIV- mothers (65.96%), although this difference was not statistically significant (p-value = 0.35). Of the 129 mothers with detectable virus, HIV+ mothers had a significantly higher EBV viral load (p-value = 0.04) than HIV- mothers (**Figure 2A**). Additionally, to determine the percentage of mothers who were shedding EBV in saliva, we analyzed DNA extracted from saliva at 6-weeks (n=149) and 10-weeks (n=159) post-partum by qPCR. We observed that at 6-weeks, the proportion of those with detectable EBV (e.g., EBV shedders) was greater in mothers that were HIV- compared to HIV+ (67.01% versus 38.46% respectively; p-value < 0.01). No significant difference was observed in the proportion at 10-weeks (74.49% for HIV- mothers and 85.71% for HIV+ mothers; p-value = 0.13). Furthermore, when we compared the EBV load in the mothers that were shedding, HIV+ mothers had significantly higher EBV viral loads compared to HIV- mothers (p-value < 0.01) at 6-weeks. There was no significant difference at 10-weeks (p-value = 0.07) (**Figure 2B**). Additionally, we analyzed changes in EBV viral load in saliva of mothers who had samples taken at both time points (6- and 10-weeks post-partum, n=116). No difference in EBV load was found when analyzing paired differences (**Supplementary Figures 1A, B**).

EBV shedding in breast milk was evaluated by qPCR using DNA extracted from breast milk at 6- weeks (n=37) and 10-weeks (n=145) post-partum. At 6-weeks the proportion of those with detectable EBV was 100% for both HIV+ and HIV- mothers. At 10-weeks, the proportion of those with detectable EBV did not differ for HIV+ mothers compared to HIV- mothers (91.19% and 86.49% respectively, p-value = 0.47). Of the mothers who were shedders (n=140), there were no significant differences in EBV viral load in breast milk samples at either 6- or 10-weeks (**Figure 2C**). When analyzing EBV viral load changes, in breast milk, of mothers who had samples taken at both time points (6- and 10-weeks post-partum, n= 29) no difference was observed (**Supplementary Figures 1C, D**).

### EBV-1 and EBV-2 Coinfection in HIV+ and HIV- Mothers

**Figure 3:**
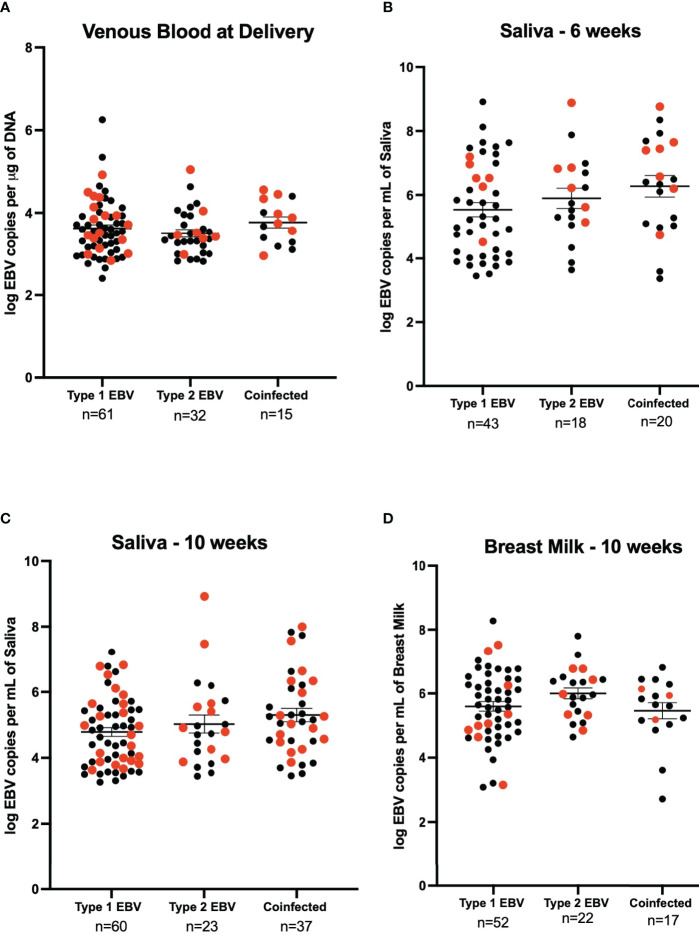
EBV viral load by EBV strain for HIV negative (black) and HIV positive (red) mothers. **(A)** Maternal venous blood cell pellet at time of delivery (n=108), 21 samples were excluded as they were not typable. We show 61 samples were EBV- 1, 44 HIV- and 17 HIV+ (3.62 EBV copies per μg of DNA), 32 were EBV- 2, 25 were HIV- and 7 HIV+ (3.50 EBV copies per μg of DNA) and 15 were coinfected, 7 HIV- and 8 HIV+ (3.77 EBV copies per μg of DNA). No significant difference was found. **(B)** Saliva 6-weeks post-partum (n=81), 4 samples were excluded as they were not typeable. EBV-1 (n=43, 37 HIV- and HIV+) had a mean viral load of 5.54 EBV copies per mL of saliva, EBV-2 (n=18, 12 HIV- and 6 HIV+) had a mean viral load of 5.90 EBV copies per mL of saliva, and coinfected (n=20, 13 HIV- and 7 HIV+) had a mean viral load of 6.26 EBV copies per mL of saliva. The difference was not found to be significantly different. **(C)** Saliva 10-weeks post-partum (n = 120), 5 samples were excluded as they were not typeable. EBV-1 (n=60, 38 HIV- and 22 HIV+) had a mean viral load of 4.78 EBV copies per mL of saliva, EBV-2 (n=23, 13 HIV- and HIV+) had a mean viral load of 5.03 EBV copies per mL of saliva, and coinfected (n=37, 21 HIV- and 16 HIV+) had a mean viral load of 5.30 EBV copies per mL of saliva. No statistical difference was found. **(D)** Breast Milk 10-weeks post-partum (n=91), 35 samples were excluded as they were not typeable. EBV-1 (n=52, 43 HIV- and 9 HIV+) had a mean viral load of 5.60 EBV copies per mL of breast milk, EBV-2 (n=22, 15 HIV- and 7 HIV+) had a mean viral load of 6.00 EBV copies per mL of breast milk, and coinfected (n=17, 14 HIV- and 3 HIV+) had a mean viral load of 5.46 EBV copies per mL of breast milk. No statistical difference was found.

We next determined the EBV strains in the saliva samples using a PCR-based assay that distinguishes EBV-1 from EBV-2 based on differences in the EBNA-3C sequence (7, 18). No statistically significant difference was observed for venous blood EBV viral load between EBV-1, EBV-2, and coinfection (3.62 log EBV copies/µg of DNA, 3.50 log EBV copies/µg of DNA, and 3.77 log EBV copies/µg of DNA respectively) (**Figure 3A**). At 6-weeks the EBV viral load in saliva was not significantly different in samples that had only EBV-1 or EBV-2 compared to coinfection (5.54 log EBV copies/mL, 5.90 log EBV copies/mL, and 6.26 log EBV copies/mL, respectively) (**Figure 3B**). Similarly, the EBV load in saliva at 10-weeks post-partum was not significantly different in terms of the EBV strain (4.78 log EBV copies/mL for EBV-1, 5.03 log EBV copies/mL for EBV-2, and 5.30 log EBV copies/mL for coinfection) (**Figure 3C**). Lastly, EBV viral load in breast milk 10-weeks post-partum was not found to differ based on EBV strain for infection (5.60 log EBV copies/mL for EBV-1, 6.00 log EBV copies/mL for EBV-2, and 5.46 log EBV copies/mL for coinfection) (**Figure 3D**). No statistically significant difference was observed when analyzing EBV strain and maternal HIV status together (stratified by EBV strain and HIV status, e.g., Type 1 & HIV Positive, Type 2 & HIV Negative, etc.; data not shown).

### EBV Viral Load in Infants Varies Based on Maternal HIV Status

**Figure 4:**
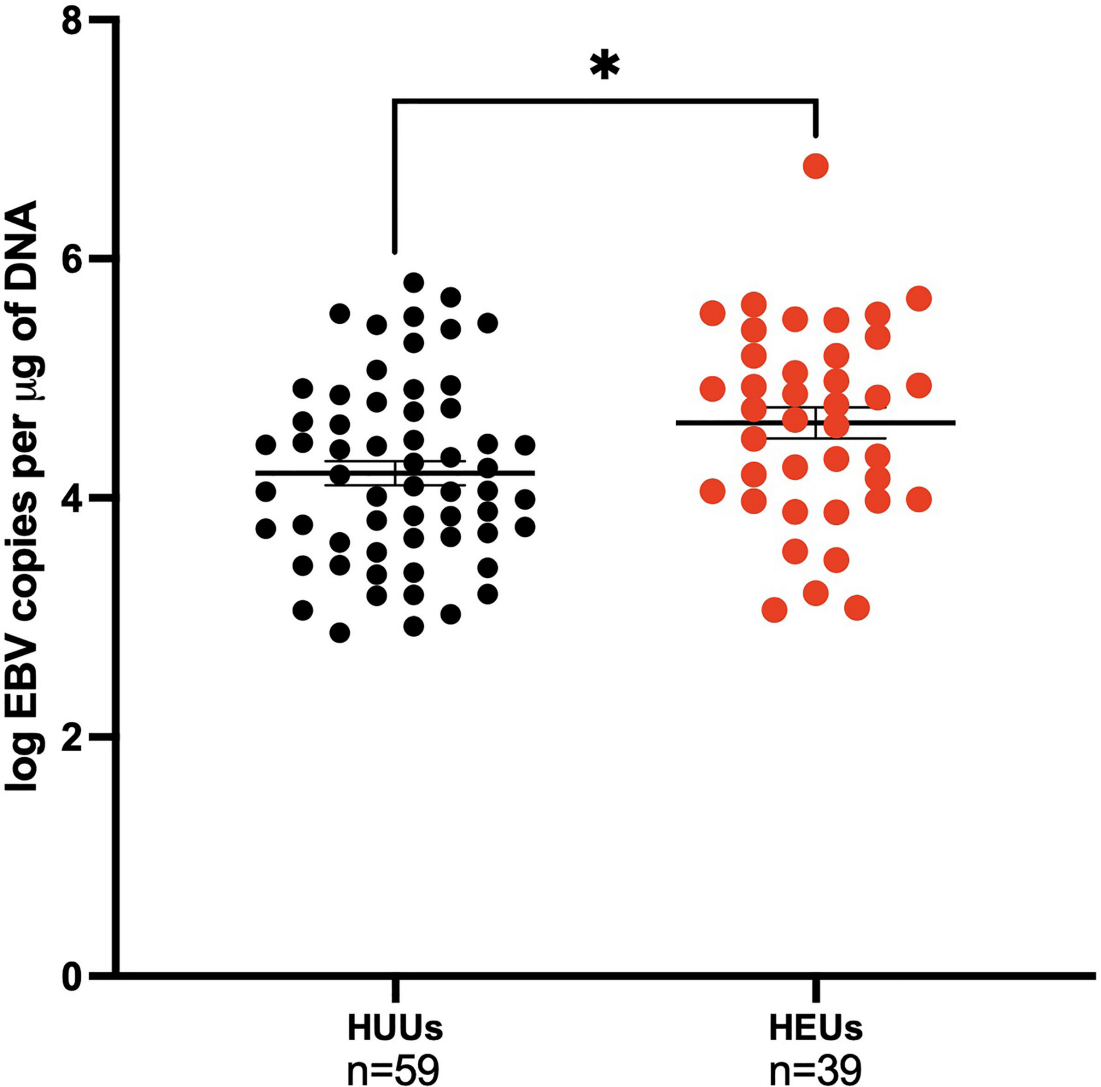
Children EBV viral load in the first positive venous blood sample. Children are categorized on maternal HIV status as HUUs (Healthy Unexposed Uninfected Children) or HEUs (Healthy Exposed Uninfected Children). HUU children have a mean of 4.21 log EBV copies per μg of DNA and 4.63 log EBV copies per μg of DNA for HEU children (p-value = 0.01). *P < 0.05.

EBV viral loads were measured by qPCR in the first EBV positive sample of infants. The mean age (SEM) for the first EBV positive sample for the HEU infants was 35 (2.99) weeks, while for the HUU infants it was 30 (3.45) weeks. At the time of first EBV DNA detection, HEU infants had significantly greater EBV viral load compared to HUU infants (4.63 log EBV copies/µg of DNA, 4.21 log EBV copies/µg of DNA, respectively; p-value = 0.01) (**Figure 4**). After adjustment for age, the conclusions were not altered (data not shown). When combined with analysis of EBV type, there were no significant pairwise differences after Bonferroni correction (stratified by EBV strain and HIV status as described previously, data not shown). Furthermore, we wanted to evaluate if mothers could serve as a source of infectious virus to their infants. We found that 43.9% of the infants had an EBV strain that was different from any of the maternal samples analyzed (e.g., venous blood, breast milk, and saliva) (**Table 2**).

**Table 2:** Potential source of mother-to-infant transmission of EBV.

Infant EBV strain	n/total samples
Same strain as Breast Milk (10-weeks post-partum)	6/98
Same strain as Venous Blood at Delivery	17/98
Same strain as Saliva (6- and 10-weeks post-partum)	17/98
Same strain as Saliva (6- and 10-weeks post-partum) Breast Milk (10-weeks post-partum)	6/98
Same strain as Saliva (6- and 10-weeks post-partum) and Venous Blood at Delivery	4/98
Same strain as all maternal samples analyzed	3/98
Different to all maternal samples analyzed	43/98
N/A – Missing information about maternal EBV	2/98

## Discussion

This study focused on HIV+ and HIV- Kenyan mothers and their role as a potential source of EBV virus transmission to their infants. We found that HIV status was not a predictor of maternal viral shedding. In fact, mothers in this study who were HIV+ were less likely to shed EBV in saliva. This is a bit paradoxical from several studies that compared EBV shedding in HIV+ vs HIV- individuals (11, 12). However, in many of those earlier studies, study participants were not on ARTs. A more recent study in Uganda did find higher prevalence of EBV shedding in HIV+ (on ARTs) vs HIV- mothers (14). Furthermore, we found that the viral load in both venous blood and 6-weeks post-partum saliva samples, of the mothers that were shedding, was higher in the HIV+ mothers compared to the HIV- mothers. Interestingly, we found a discordance in two compartments where EBV is maintained as reflected by the differences in detection of EBV in breast milk as compared to venous blood. Furthermore, we did not observe a correlation between having detectable viral DNA in venous blood to shedding of virus in saliva or breast milk. This is consistent with the studies of Labo et al. (13) and suggests that control of virus replication in different cellular compartments is independently regulated.

Consistent with our previous observation of the presence of both EBV-1 and EBV-2 in this region (18), we found both EBV strains in maternal samples and co-infection in some samples, regardless of HIV status. We did not observe differences in viral load based on the EBV strain. Interestingly, we found EBV-2 in 40.8% of venous blood from infants, regardless of maternal HIV status; but only in 29.6% of maternal venous blood at delivery, 22.2% of 6-weeks post-partum saliva, 19.2% in 10-weeks post-partum saliva, and 24.2% in 10-weeks post-partum breast milk.

A particularly novel finding from this study is infant EBV viral load relative to maternal HIV status. We found that HEU infants have a significantly higher EBV viral load than HUU infants at time of first detection. It has been previously suggested that maternal immunosuppression may impair placental transfer of EBV-specific antibodies to the infant, making HEU infants more vulnerable to EBV infection (4, 27). To our knowledge, this is the first study comparing HEU vs HUU in terms of EBV infection, in which neonatal exposure to maternal HIV was associated with higher EBV viral loads. These findings are consistent with our previous observation (3) where we reported that in a region where there is a high incidence of eBL infants were infected with EBV earlier in life and had higher EBV viral loads and with those of Slyker et al, (4) who found higher incidence of EBV infection in very young HIV+ infants. Together with those studies, this led us to hypothesize that higher viral loads of EBV during primary infection may be an important risk factor for the development of EBV-related malignancies, including but not limited to BL. The finding that 43.9% of the infants had an EBV strain that was different than the maternal strain suggests that mothers are more than a source of infectious virus, are determinants of the infant control of EBV infection and that maternal HIV status plays a major role in this.

The main strength of our study is the longitudinal assessment of study participants with enrollment of mothers and their infants at time of birth. There are a few limitations of this study. First, the timing of viral shedding, inter-individual variability could account for differences between the HIV+ and HIV- mothers. In addition, while all of the HIV+ mothers in our study were treated for HIV through pregnancy and beyond, we did not collect data on CD4 count or HIV viral load.

In conclusion, infants born to mothers with HIV had higher EBV viral loads at the time of primary infection compared to infants born to HIV-negative mothers. Whether this reflects higher levels of shedding of EBV, either in saliva or breast milk, in the mother, or impairment of transfer of maternal antibodies to infants, or an effect on *in utero* exposure to HIV resulting in impaired immunity in their infant remains unknown. The early life infection of EBV and higher viral loads in these HEU infants potentially puts them at risk for development of BL.

## Data Availability Statement

The raw data supporting the conclusions of this article will be made available by the authors, without undue reservation.

## Ethics Statement

The studies involving human participants were reviewed and approved by Scientific and Ethical Review Unit (SERU) at the Kenya Medical Research Institute (KEMRI), Colorado Multiple Institutional Review Board (COMIRB), and SUNY Upstate Medical University (where the study was initiated) ethical review boards. Written informed consent to participate in this study was provided by the participants’ legal guardian/next of kin.

## Author Contributions

GS-R designed research, performed research, analyzed data, wrote the paper. SO designed the research, performed research and wrote the paper. ID designed the research, performed research and wrote the paper. CJ analyzed data, wrote the paper. KS analyzed data, wrote the paper. AD designed research, analyzed data and wrote the paper. RR designed research, analyzed data and wrote the paper. All authors contributed to the article and approved the submitted version.

## Funding

This work was supported by the National Institutes of Health [grant numbers AI098511 (AD), CA102667 (RR) AI1141531(RR), D43 153707 (SO, ID)].

## Conflict of Interest

The authors declare that the research was conducted in the absence of any commercial or financial relationships that could be construed as a potential conflict of interest.

## Publisher’s Note

All claims expressed in this article are solely those of the authors and do not necessarily represent those of their affiliated organizations, or those of the publisher, the editors and the reviewers. Any product that may be evaluated in this article, or claim that may be made by its manufacturer, is not guaranteed or endorsed by the publisher.
