# Supplementary figures and images for: Maternal HIV Infection as a Risk Factor for Primary Epstein-Barr Virus Infection in Kenyan Infants
Source: Front Oncol. 2022 Jan 12;11:805145. doi: 10.3389/fonc.2021.805145 (PMC8790250; doi:10.3389/fonc.2021.805145)

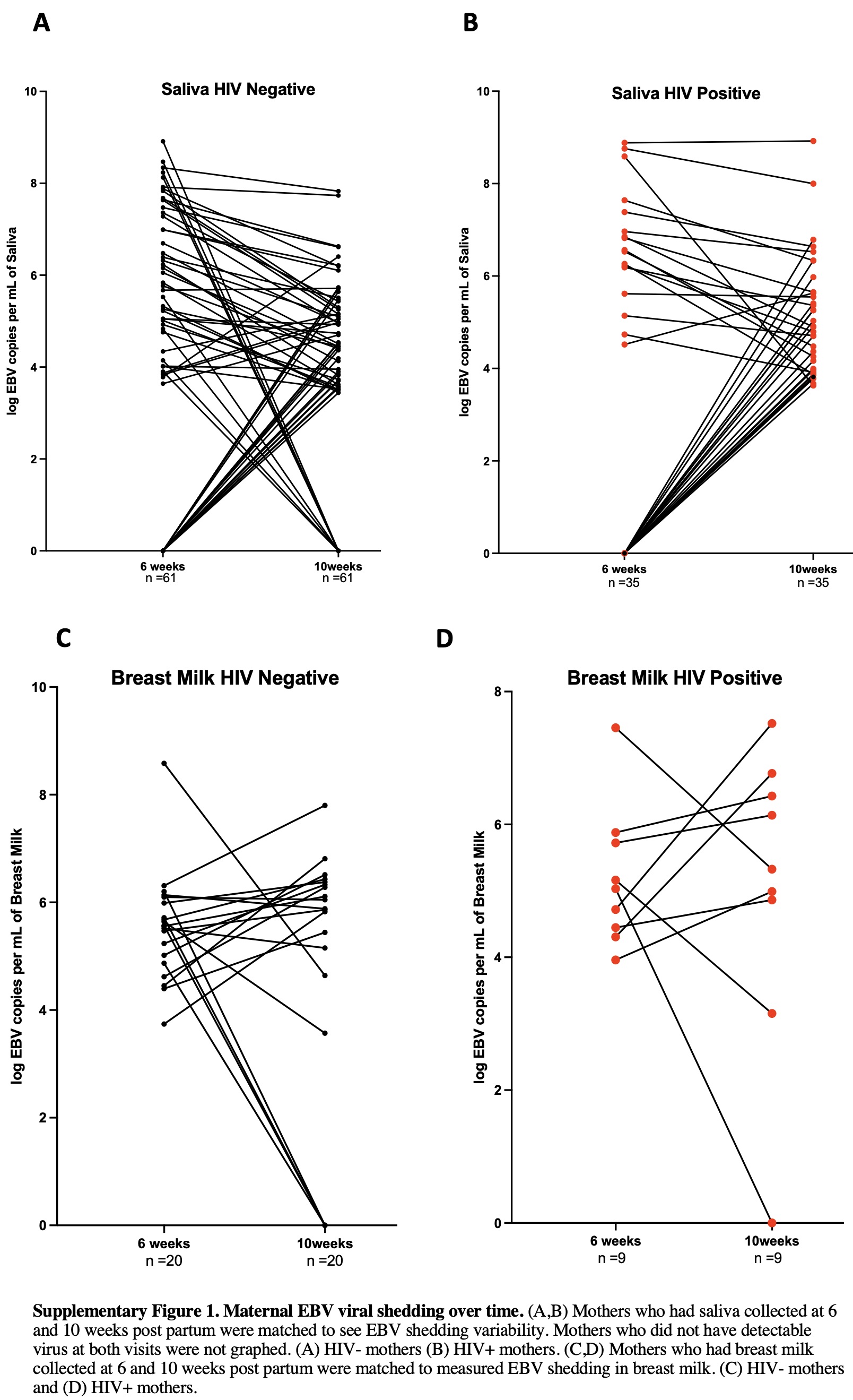

Supplement: Supplementary file 1 [file Image_1.jpg]
